# Job satisfaction and its demographic, occupational, and mental health determinants among community pharmacists

**DOI:** 10.1371/journal.pone.0341726

**Published:** 2026-02-12

**Authors:** Anan S. Jarab, Ahmad Z. Al Meslamani, Walid Al-Qerem, Hamza Jarab, Eman Merghani Ali Mohammed, Hebatallah Ahmed Mohamed Moustafa, Abdallah Y. Naser, Yazid N. Al Hamarneh, Salahdein Aburuz

**Affiliations:** 1 Department of Clinical Pharmacy, Faculty of Pharmacy, Jordan University of Science and Technology, Irbid, Jordan; 2 College of Pharmacy, Al Ain University, Abu Dhabi, United Arab Emirates; 3 Department of Pharmacy, Faculty of Pharmacy, Al-Zaytoonah University of Jordan, Amman, Jordan; 4 Faculty of Medicine, Jordan University of Science and Technology, Irbid, Jordan; 5 Clinical Pharmacy Department, College of Pharmacy, Jazan University, Jazan, Saudi Arabia; 6 Clinical pharmacy and pharmacy practice department, Faculty of Pharmacy, Badr University in Cairo, Cairo, Egypt; 7 Department of Applied Pharmaceutical Sciences and Clinical Pharmacy, Faculty of Pharmacy, Isra University, Amman, Jordan; 8 Department of Pharmacology, Faculty of Medicine and Dentistry, University of Alberta, Edmonton, Canada; 9 Department of Pharmacology and Therapeutics, College of Medicine and Health Sciences, United Arab Emirates University, Al Ain, United Arab Emirates; University of Petra (UOP), JORDAN

## Abstract

**Background:**

Job satisfaction among community pharmacists influences service quality, patient outcomes, and workforce stability, yet evidence from Jordan is limited and fragmented.

**Objectives:**

To determine the overall level of job satisfaction among Jordanian community pharmacists and examine its associations with demographic, occupational, and mental-health factors.

**Methods:**

A cross-sectional study was among licensed community pharmacists working in independent and chain pharmacies across Jordan. Data were collected using a validated, self-administered online questionnaire distributed through convenience sampling. The survey included items on demographic and occupational characteristics, as well as three validated mental health scales: the Generalized Anxiety Disorder-7 (GAD-7), Patient Health Questionnaire-9 (PHQ-9), and Perceived Stress Scale-10 (PSS-10). Job satisfaction was assessed using a single-item global question. A binary logistic regression analysis was conducted to explore the variables associated with job satisfaction among participants.

**Results:**

Among 385 respondents, 257 (66.8%) pharmacists reported being satisfied with their job. Satisfied pharmacists handled more patients per shift (median 150 [IQR 100–250] vs 150 [40–200]; p = 0.050), filled more prescriptions (60 [30–90] vs 50 [24–90]; p = 0.042), and dispensed more medications (200 [100–300] vs 175 [57.5–300]; p = 0.028). In multivariable analysis, fixed-evening (AOR 0.46, 95% CI 0.22–0.95) and flexible shifts (AOR 0.41, 95% CI 0.19–0.89) reduced satisfaction odds, whereas each additional patient handled slightly increased satisfaction odds (AOR 1.004, 95% CI 1.001–1.008).

**Conclusion:**

Mental-health symptom burden and most demographic factors are unrelated to satisfaction, whereas workload patterns, particularly shift timing and patient volume, exert significant influence. Optimising shift assignments and staffing to balance high patient engagement with manageable hours may enhance pharmacist retention and care quality.

## Introduction

Job satisfaction is defined as “an employee’s positive reaction towards his/her work” [1]. In the context of pharmacy, job satisfaction among pharmacists has important implications. It affects quality of pharmaceutical care and patient health outcomes [2,3]. Healthcare practitioners with high job satisfaction achieve better unit-level outcomes, including increased productivity, higher patient satisfaction, and greater organisational profitability [4,5]. Due to high work load and different working shifts, healthcare professionals become prone to different types of psychological problems [6–8]. At the individual level, it correlates with higher performance, stronger organisational citizenship behaviours, and reduced unproductive work [9,10]. This relationship underscores that job happiness is vital to an organisation’s performance and survival, not only to employee well-being. Because happy pharmacists are more focused, productive, and less likely to resign, the healthcare system can deliver better patient care [2,3].

Community pharmacists showed different levels of job satisfaction across countries and even within the same country. In Saudi Arabia, the level of job satisfaction among pharmacists ranged from 22.4% to 76.7% [11,12]. In Lebanon, around half of participated pharmacists showed job satisfaction [13], while rates ranged from 41.4% in Nigeria [14], to 88.1% in Malaysia [15], and 57% of pharmacists in Ireland reported being satisfied with their job [16]. Workload, compensation, opportunities for career advancement, and recognition were all consistently cited as key factors affecting pharmacists’ satisfaction with their job. For example, Al Khalidi and Wazaify reported that most hospital and community pharmacists in Jordan consider prolong work hours and lack of incentives have substantial influence on their level of satisfaction [17]. Poorer mental health including signs of depressions, stress, and anxiety were also associated with job dissatisfaction [18–20].

In Jordan, recent studies show diverse but concerning results for community pharmacists. A 2024 survey of 400 pharmacists reported a mean Copenhagen Burnout Inventory score of 49.7 ± 16.7, with 43% meeting the high-burnout threshold [21]. Another survey of 693 pharmacy-degree holders found an “ambivalent” mean Job Satisfaction Scale score of 126.9/216 [22]. Another study showed that 50.5% of pharmacists had recently managed patients with suicidal thoughts, yet only 17.7% felt confident recognising warning signs, highlighting cultural taboos, limited training, and a psychological burden that may exacerbate burnout [23].

Understanding Jordanian community pharmacists’ job satisfaction is essential for health-workforce planning and patient care. Because existing evidence is based on older or indirect investigations, it may not reflect current conditions. International and regional data indicate that several pressures influence pharmacists, but their effects in Jordan remain unclear. Changes in remuneration, professional support, and pandemic-related workloads may have further altered the landscape. This research addresses the knowledge gap by analysing job satisfaction among Jordanian community pharmacists and its determinants. Therefore, this study aims to determine the overall level of job satisfaction among Jordanian community pharmacists and to assess its associations with demographic, occupational, and mental health factors.

## Materials and methods

### Study design, setting, and participants

This cross-sectional study targeted licensed pharmacists working in independent and chain community pharmacy settings across Jordan between January and May 2025. Pharmacists eligible for participation were those who had graduated from universities accredited by the Jordanian Ministry of Higher Education and were registered as community pharmacists with the Jordanian Ministry of Health. Pharmacists with less than six months of work experience were excluded to ensure that participants had sufficient exposure to typical workplace conditions. Data collection was carried out using a validated electronic questionnaire distributed via Google Forms. A convenience sampling strategy was employed, and research team members based in different geographic regions helped promote the survey through direct outreach and professional social media networks, such as WhatsApp and Facebook groups for pharmacists. Before starting the survey, participants were informed about the aim of the study and assured that their participation was voluntary, anonymous, and confidential. Electronic informed consent was obtained from all participants prior to accessing the questionnaire. On average, the survey required 10–12 minutes to complete and submitted responses were reviewed for completeness before analysis.

### Ethical approval

Ethical approval for this study was obtained from the Institutional Review Board (IRB) and the Deanship of Research at Jordan University of Science and Technology (Reference No. 38/176/2024) on December 8, 2024. Participants who chose to take part in the study provided informed consent before proceeding.

### Study instruments

The study questionnaire consisted of several sections designed to assess demographic, occupational, and mental health variables relevant to pharmacists’ job satisfaction. The first section covered demographic and work-related characteristics, including age, sex, level of education, type of pharmacy (independent or chain), employment status (full-time or part-time), years of experience, work schedule, and frequency of working overtime. Additional questions explored participants’ perceptions of salary adequacy, access to job benefits (e.g., medical insurance or retirement plans), the level of societal recognition pharmacists receive, career advancement opportunities, and concerns regarding unethical competition and workforce oversupply. Job satisfaction was measured using a single global item: *“Are you satisfied with your job as a pharmacist?”*, with response options of “Yes” or “No.”

Mental health symptoms were assessed using three validated instruments. The Generalized Anxiety Disorder-7 (GAD-7) is a 7-item scale measuring the frequency of anxiety symptoms over the previous two weeks using a four-point Likert scale ranging from 0 (“not at all”) to 3 (“nearly every day”), with total scores ranging from 0 to 21 and severity classified as mild (5–9), moderate (10–14), or severe (15–21); a score ≥10 was used to indicate clinically significant anxiety [24]. The Patient Health Questionnaire-9 (PHQ-9) assesses depressive symptoms using 9 items scored similarly on a 0–3 scale, with total scores from 0 to 27; severity was categorized as mild (5–9), moderate (10–14), moderately severe (15–19), or severe (20–27), and a score ≥10 indicated clinically relevant depression [25]. The Perceived Stress Scale-10 (PSS-10) evaluates perceived stress over the past month using 10 items scored from 0 (“never”) to 4 (“very often”), producing a total score from 0 to 40, with stress levels categorized as low (0–13), moderate (14–26), and high (27–40) [26]. All three scales are widely used, psychometrically sound tools that have been validated in Arabic-speaking populations. In the current study, internal consistency was acceptable for all scales, with Cronbach’s alpha values of 0.81 for GAD-7, 0.86 for PHQ-9, and 0.78 for PSS-10. Each instrument was selected based on its established psychometric properties. Face validity was further confirmed through a pilot test conducted with 15 community pharmacists to evaluate item clarity, structure, and overall flow of the questionnaire. Data collected during the pilot phase were excluded from the final analysis.

### Sample size calculation

The formula: n = (Z² × p × (1 – p))/ d² was used for sample size calculation [27]. In this formula, *p* represents the estimated proportion of the population, which we set at 0.5 to reflect the highest possible variability. The margin of error (d) was set at 0.05, and we used a Z-value of 1.96, corresponding to a 95% confidence level. Based on these parameters, the minimum required sample size was calculated to be 385 participants.

### Statistical analysis

Data analysis was performed using the SPSS V26.0. The primary outcome was overall job satisfaction (Yes/No). Categorical variables, such as sex, age group, degree, employment type, work-schedule categories, perceptions and attitudes, were analyzed as nominal variables. Continuous or ordinal workload metrics, such as average patients, prescriptions, medications per shift and weekly working hours were analyzed on their original scales. Descriptive statistics were reported as counts (percentages) for categorical variables and medians with interquartile ranges (IQRs) for non-normally distributed continuous variables.

Associations between categorical predictors and job satisfaction were evaluated with Pearson’s chi-square test; Fisher’s exact test was planned if expected cell counts were <5. For continuous/ordinal workload metrics by job-satisfaction status, the Mann–Whitney U test was used given non-normality. Results were presented as medians (IQR) with exact or asymptotic p-values as appropriate. A p value less than 0.05 defined statistical significance.

To identify independent predictors of job satisfaction, a binary logistic regression with job satisfaction (Yes = 1, No = 0) as the dependent variable was conducted. Candidate covariates included highest educational qualification (reference: PhD), employment status (reference: part-time), typical work schedule (reference: rotating), perceived sufficiency of benefits (reference: Yes), and continuous workload measures of average weekly hours, average patients, prescriptions, and medications per shift; modeled per one-unit increase, as reported). Categorical predictors were dummy coded with the reference categories matching Table 4. Variables were retained a priori if theoretically relevant and/or showed bivariable associations at p < 0.20. Adjusted odds ratios (AORs) with Wald 95% confidence intervals (CIs) and p-values are reported.

## Results

In this study, 385 community pharmacists completed the questionnaire, of which 283 (73.5%) were females, 165 (42.9%) had from 5 to 10 years of experience, and 245 (63.6%) worked in independent pharmacies (Table 1). Among the study participants, 257 (66.8%) were satisfied with their job as pharmacists. Sex, age, years of experience, education level, work position (full- vs. part-time), pharmacy type (chain vs. independent), work schedule, and overtime practice did not significantly affect job satisfaction (all p > 0.05).

**Table 1:** Sociodemographic and job-related characteristics of pharmacists and their association with job satisfaction (N = 385).

Item	Total, n (%)	Satisfied with your job as a pharmacist		
		Yes	No	P value
Sex				0.475
Female	283 (73.5%)	186 (65.7%)	97 (34.3%)	
Male	102 (26.5%)	71 (69.6%)	31 (30.4%)	
Age (years)				0.681
22-26	92 (23.9%)	58 (63.0%)	34 (37.0%)	
27-34	229 (59.5%)	156 (68.1%)	73 (31.9%)	
≥ 35	64 (16.6%)	43 (67.2%)	21 (32.8%)	
Years of experience				0.684
< 5	142 (36.9%)	95 (66.9%)	47 (33.1%)	
5-10	165 (42.9%)	113 (68.5%)	52 (31.5%)	
> 10	78 (20.3%)	49 (62.8%)	29 (37.2%)	
Educational degree				0.241
Bachelor’s in pharmacy (BPharm)	199 (51.7%)	129 (64.8%)	70 (35.2%)	
Doctor of pharmacy (PharmD)	141 (36.6%)	102 (72.3%)	39 (27.7%)	
Master’s	39 (10.1%)	22 (56.4%)	17 (43.6%)	
PhD	6 (1.6%)	4 (66.7%)	2 (33.3%)	
Current employment				0.172
Full-time	238 (61.8%)	165 (69.3%)	73 (30.7%)	
Part-time	147 (38.2%)	92 (62.6%)	55 (37.4%)	
Type of pharmacy				0.425
Chain	140 (36.4%)	97 (69.3%)	43 (30.7%)	
Independent	245 (63.6%)	160 (65.3%)	85 (34.7%)	
Work shifts				0.050
Daytime	116 (30.1%)	79 (68.1%)	37 (31.9%)	
Evening	47 (12.2%)	27 (57.4%)	20 (42.6%)	
Night	24 (6.2%)	19 (79.2%)	5 (20.8%)	
Rotating shifts	160 (41.6%)	113 (70.6%)	47 (29.4%)	
Flexible hours	38 (9.9%)	19 (50. %)	19 (50.0%)	
Working overtime				0.787
Yes	96 (24.9%)	63 (65.6%)	33 (34.4%)	
No	289 (75.1%)	194 (67.1%)	95 (32.9%)	

Most of the 385 pharmacists surveyed felt adequately paid (322/385, 83.6%), yet only a minority believed their position offered sufficient benefits, such as medical insurance or retirement plans (144/385, 37.4%); about two-thirds felt the profession was undervalued by the community (Table 2). There were no significant differences in job satisfaction across perceptions of adequate pay, sufficient benefits, feeling undervalued by the community, limited career-growth opportunities, unethical competition, mismatched undergraduate expectations, or concern about an oversupply of pharmacy graduates.

**Table 2:** Associations between job satisfaction and pharmacists’ perceptions of job benefits, professional stressors, and market pressures (N = 385).

Variable	Total, n (%)	Satisfied with your job as a pharmacist		
		Yes	No	P value
Receiving rewording salary				0.372
Yes	322 (83.6%)	218 (67.7%)	104 (32.3%)	
No	63 (16.4%)	39 (61.9%)	24 (38.1%)	
The job provides sufficient benefits (medical insurance, retirement plans, etc..)				0.189
Yes	144 (37.4%)	102 (70.8%)	155 (64.3%)	
No	241 (62.6%)	42 (29.2%)	86 (35.7%)	
Feeling the community undervalues the role pharmacists play.				0.495
Yes	268 (69.6%)	176 (65.7%)	92 (34.3%)	
No	117 (30.4%)	81 (69.2%)	36 (30.8%)	
Feeling career growth pathways within pharmacy are limited.				0.538
Yes	275 (71.4%)	181 (65.8%)	94 (34.2%)	
No	110 (28.6%)	76 (69.1%)	34 (30.9%)	
Believing in the existence of unethical competition from other pharmacists				0.439
Yes	171 (44.4%)	111 (64.9%)	60 (35.1%)	
No	214 (55.6%)	146 (68.2%)	68 (31.8%)	
Perceiving a clear gap between the responsibilities of community pharmacist and the expectations formed in pharmacy school.				0.890
Yes	278 (72.2%)	185 (66.5%)	93 (33.5%)	
No	107 (27.8%)	72 (67.3%)	35 (32.7%)	
Concerned about the increasing number of pharmacy graduates in the country				0.712
Yes	269 (69.9%)	178 (66.2%)	91 (33.8%)	
No	116 (30.1%)	79 (68.1%)	37 (31.9%)	

**Fig 1:**
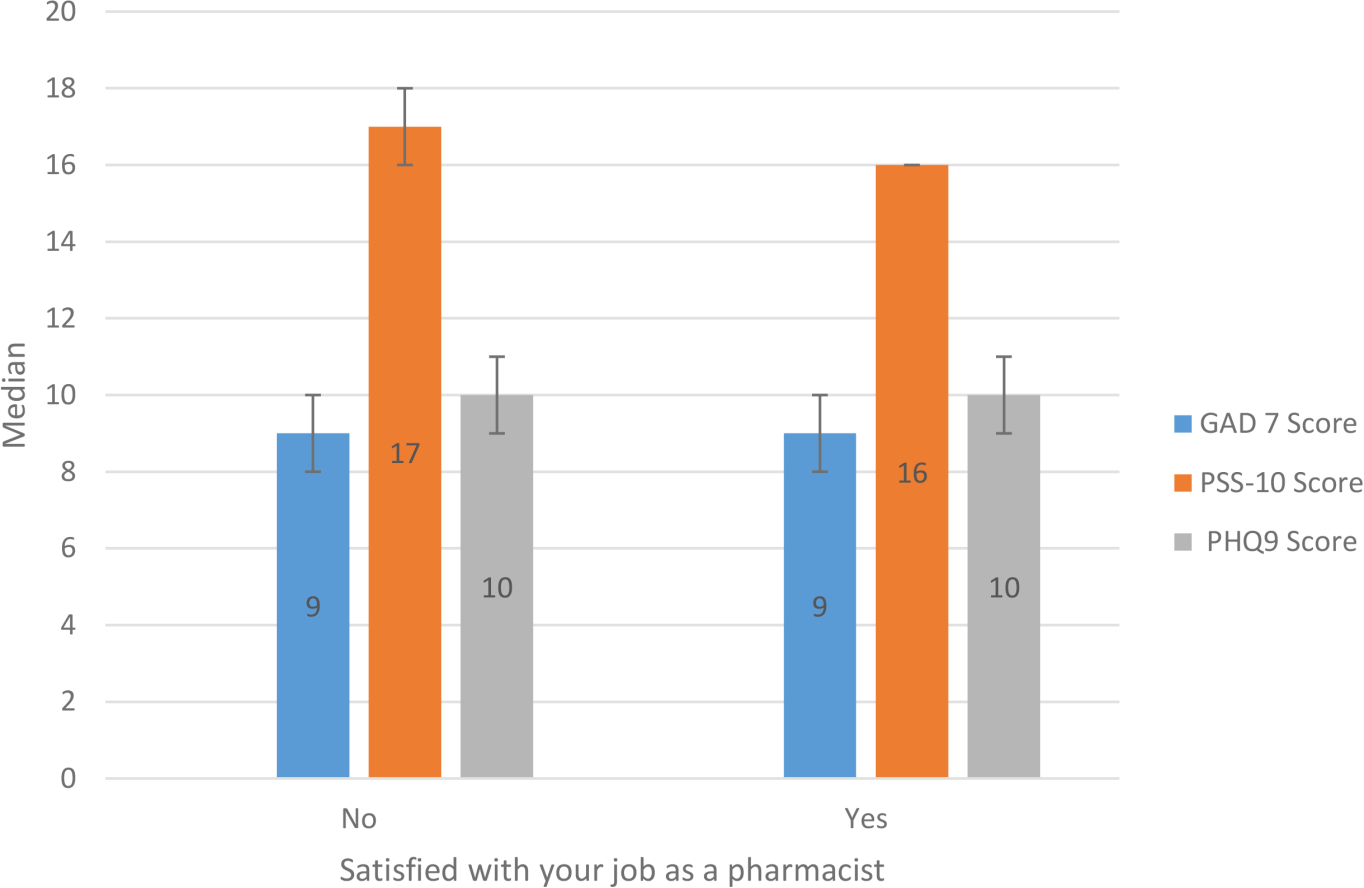
Relationship between job satisfaction and mental-health scores among community pharmacists (N = 391). (*Mann–Whitney U tests detected no statistically significant differences between the two groups for any of the three outcomes (all p > 0.05).).*

Median anxiety (GAD-7), perceived stress (PSS-10), and depression (PHQ-9) scores for pharmacists who were satisfied with their jobs and those who were not, were 9 vs 9, 16 vs 17, and 10 vs 10, respectively (Fig 1). job satisfaction was not associated with mental-health symptom burden (all p value >0.05).

Pharmacists who reported being satisfied handled a median of 150 patients per shift (IQR = 100–250) versus 150 patients (40–200) among the dissatisfied (p = 0.05) (**Table 3**).They also filled more prescriptions, 60 (30–90) versus 50 (24–90) (p = 0.042) and dispensed more medications, 200 (100–300) versus 175 (57.5–300) (p = 0.028) Years of professional experience did not differ meaningfully between groups, 42 years (36–48) for the satisfied versus 48 years (36–49) for the dissatisfied (p = 0.052).

**Table 3:** Workload and experience metrics stratified by overall job satisfaction among community pharmacists.

Item	Satisfied with your job as a pharmacist		
	Yes, median (IQR)	No, Median (IQR)	P value
The average number of patients you handled per shift	150.0 (100.0-250.0)	150.0 (40.0-200.0)	**0.050**
The average number of prescriptions filled per shift	60.0 (30.0-90.0)	50.0 (24.0-90.0)	**0.042**
The average number of medications you dispensed per shift	200.0 (100.0-300.0)	175.0 (57.5-300)	**0.028**
How many years of work experience do you have?	42.0 (36.0-48.0)	48.0 (36.0-49.0)	0.052

P-values were computed with the two-tailed Mann–Whitney U test, which compares the distributions of two independent samples when normality cannot be assumed; statistical significance was defined as p < 0.05. Bold indicates significant results.

**Table 4:** Logistic regression analysis of variables associated with job satisfaction among community pharmacists.

Independent variable (reference = 1.00)	AOR	95% CI	P value
**Highest educational qualification**			
PhD in pharmacy (Ref)	1.00	–	–
Bachelor’s degree (BPharm)	0.35	0.06–2.17	0.257
Doctor of Pharmacy (PharmD)	0.61	0.10–3.80	0.594
Master’s degree	0.27	0.04–1.79	0.174
**Current employment**			
Part-time (Ref)	1.00	–	–
Full-time	1.20	0.73–1.98	0.471
**Typical work schedule**			
Rotating (Ref)	1.00	–	–
Fixed day	0.87	0.50–1.50	0.610
Fixed evening	0.46	0.22–0.95	**0.035**
Flexible	0.41	0.19–0.89	**0.023**
Night shift	1.64	0.54–4.93	0.381
**Perceived sufficiency of benefits**			
Yes (Ref)	1.00	–	–
No	0.78	0.48–1.26	0.312
Continuous workload measures*			
Average working hours/ week^α^	1.001	0.990–1.012	0.867
Average patients handled/ shift^α^	1.004	1.001–1.008	**0.019**
Average prescriptions filled/ shift^α^	0.996	0.991–1.002	0.216
Average medications dispensed/ shift^α^	1.000	0.999–1.002	0.611

* Continuous predictors are expressed per one-unit increase. ^α^*Units: hours, patients, prescriptions, or medications, respectively. Adjusted odds ratios (AORs) are exponentiated logistic coefficients; 95% confidence intervals (CIs) and p values derive from Wald statistics.*

Compared with pharmacists on rotating shifts, those on fixed-evening schedules had markedly lower odds of being satisfied (AOR 0.46; 95% CI 0.22–0.95; p = 0.035), and those on flexible hours showed a similar reduction (AOR 0.41; 95% CI 0.19–0.89; p = 0.023) (Table 4). In contrast, each additional patient handled per shift conferred a small but significant increase in satisfaction odds (AOR 1.004; 95% CI 1.001–1.008; p = 0.019). Given the unit of measurement, the effect size is minimal at the individual-patient level (almost 0.4% higher odds per additional patient) and should not be over-interpreted as a large practical difference.

## Discussion

This study aimed to evaluate job satisfaction among community pharmacists in Jordan and identify how demographic factors, work characteristics, and mental health relate to their satisfaction. Understanding these determinants is important because pharmacist satisfaction can influence turnover [28], service quality, and patient care outcomes [14,29].

In this study, females represented 73.5% of respondents. This aligns with national health workforce reporting in Jordan; for example, the High Health Council’s National Human Resources for Health Strategy for Jordan (2018–2022) reports that 75.3% of Ministry of Health pharmacists are female [30].

In this study, around two-thirds of the pharmacists were satisfied with their job, which is relatively comparable to that found among Jordanian pharmacists more than a decade ago [17]. Such cross-study variability is not unique to Jordan. For example, a study from Saudi Arabia indicates substantially lower satisfaction, with fewer than one-quarter of pharmacists expressing contentment with their work [11]., By contrast, another 2020 Saudi study observed a much higher 76.7% job satisfaction rate among community pharmacists [12]. Globally, satisfaction levels varied from 41.4% in Nigeria [14], to 88.1% in Malaysia [15]. This cross-national variability likely reflects factors unique to each country, such as heavier workloads, lower remuneration, and differing levels of professional autonomy. Within a single country, the discrepancies we reported can arise from variations in sampling frames (e.g., chain versus independent pharmacies), survey timing, and the measurement instruments employed.

The findings of this study showed thar pharmacists working fixed-evening shifts had significantly lower odds of job satisfaction than those with regular day shifts. This result aligns with evidence that irregular or night schedules can negatively impact healthcare workers’ well-being and attitudes. For example, a recent study in Jordan found that night-shift health workers reported more intent to leave and overall lower job satisfaction [31]. Qualitative research explained this as pharmacists have complained that “always night shift” duties harm their health and morale [32]. We expect that evening or constantly changing shifts would undermine satisfaction, likely due to disrupted work-life balance, fatigue, and social isolation.

Interestingly, our findings show higher odds of satisfaction among pharmacists who handled a greater number of patients per shift. At first glance, one might expect heavy patient load to increase stress and lower satisfaction; indeed, excessive workload is often cited as a cause of dissatisfaction among pharmacists in the Middle East [32]. However, However, two factors may help explain this counterintuitive result. First, rising patient traffic may increase pharmacy income, thereby enhancing pharmacists’ job stability, reputation, and pay. Second, serving numerous patients can help pharmacists feel that they are fully using their clinical skills and making a meaningful difference, which may offset the stress of a heavy workload. Consistent with this, a Saudi survey found that community pharmacists who treated more than 30 patients per day were more satisfied with their profession [11]. From the standpoint of occupational stress, patient volume is an objective work requirement that, depending on the resources and incentives available, may either improve motivation or worsen well-being. According to the Job Demands–Resources (JD-R) framework, demands, such as high work pressure, are not intrinsically harmful; rather, they become stressful when sustained effort is not accompanied by sufficient recovery and resources [33]. On the other hand, resources, such as autonomy, feedback, support, and adequate staffing or technology, can mitigate the negative effects of high demands while fostering engagement and professional development. Particularly in community pharmacies, workload involves both quantitative and subjective aspects, and the subjective experience is strongly associated with pharmacist outcomes such as burnout and job satisfaction [34]. Internationally, qualitative data from England show that increasing workloads in community pharmacies are frequently perceived as causing stress and lowering job satisfaction [35].

The findings of this study did not find significant associations between pharmacists’ job satisfaction and their mental health scores (anxiety, stress, or depression), which contrasts most of the studies in the literature. In Vietnam, Increased depression (AOR = 0.441) was linked to job dissatisfaction among pharmacists [18]. Similarly, there was a significant inverse association between occupational stress and job satisfaction among pharmacists in Saudi Arabia (β = −0.456) [19]. Furthermore, Greek community pharmacists who reported higher emotional exhaustion and lower personal accomplishment were significantly correlated with poorer SF-36 mental-health scores (e.g., vitality r = –0.15, emotional well-being r = –0.24; all p < 0.01) [20]. The absence of a link in our sample may have several causes. Most pharmacists in the study may have experienced only moderate anxiety or depression that did not affect their work attitude. Pharmacy staff might cope with mild stress and still feel satisfied at work if they regard these challenges as part of the job. Job satisfaction could also be driven more by external factors—such as pay, workload, and recognition, than by internal emotional symptoms. Finally, because the study was cross-sectional, dissatisfied pharmacists may have already left the workplace or sought mental-health care, thereby weakening any observable relationship.

To sum up, this study provides up to date evidence from Jordan that, although personal mental-health metrics may not directly predict community pharmacists’ job satisfaction, work-schedule and workload factors can have a substantial impact. These results contribute to the literature by highlighting context-specific factors and indicating that, to increase job satisfaction, pharmacy employers and legislators should prioritise optimising shift assignments, staffing levels, and opportunities for patient involvement. By addressing these issues, pharmacy practices may be able to retain satisfied pharmacists, thereby enhancing patient outcomes and service quality in local communities. However, to fully capturing the picture, future research should use a longitudinal, multi-centre cohort design that follows community pharmacists over several years, systematically measuring stressors, mental-health status, and job-satisfaction scores to clarify causal pathways and temporal trends.

### Study limitations

It is important to recognise the many limitations of this study. Because of its cross-sectional design, which inherently restricts causal inference, we cannot be sure whether these characteristics affected job satisfaction or whether, conversely, happier pharmacists opted out of crowded workplaces. Secondly, because all information was self-reported, response bias may have occurred. For instance, participants might overestimate job satisfaction or under-report mental-health problems owing to stigma Because self-administered self-report scales were used to measure anxiety, depression, and stress, responses may have been influenced by social desirability and under-reporting, especially in environments where mental health stigma is still prevalent. This could have biased symptom estimates downward and attenuated any true association with job satisfaction.

Additionally, the sample was limited to community pharmacists in Jordan and may not be entirely representative of all pharmacists in the country; generalisability may be affected by geographical differences or non-respondent characteristics. Furthermore, the GAD-7, PHQ-9, and PSS-10 were screening instruments rather than clinical diagnostic tools, and most respondents recorded very low scores, which may have reduced our ability to detect associations with satisfaction. Furthermore, selection bias due to social media usage is feasible, since recruitment depended extensively on online distribution (e.g., WhatsApp/Facebook groups). Individuals who are less active online, typically older or more senior professionals, may be systematically underrepresented in online recruitment, which might have affected the observed satisfaction distribution and hampered generalisability.

Lastly, some of the observed relationships may have been distorted by unmeasured factors such as exact working hours, pay, or the number of support staff. Notwithstanding these drawbacks, the study provides valuable insights; nevertheless, the findings should be interpreted cautiously and confirmed by further qualitative or longitudinal research.

## Conclusion

This study sample of community pharmacists in Jordan reported an overall positive job satisfaction, which appeared unrelated to their demographic characteristics, education, or perceptions of pay and benefits. Anxiety, stress, and depressive symptoms showed no clear connection to how content they felt at work. Instead, satisfaction was significantly associated with workload patterns, with higher patient and prescription volumes fostering greater fulfilment, whereas fixed-evening or flexible schedules were linked to poorer satisfaction.

## Supporting information

S1 Checklist Strobe. (DOC)

S2 Questionnaire Stude Questionnaire. (DOCX)

S3 Data Study Data. (XLSX)

S4 File Inclusivity-in-global-research-questionnaire. (DOCX)
