# Supplementary material for: Job satisfaction and its demographic, occupational, and mental health determinants among community pharmacists
Source: PLoS One. 2026 Feb 12;21(2):e0341726. doi: 10.1371/journal.pone.0341726 (PMC12900340; doi:10.1371/journal.pone.0341726)
Supplement: S2 Questionnaire — (DOCX) [file pone.0341726.s002.docx]

**Job Satisfaction and Its Demographic, Occupational, and Mental Health Determinants Among Community Pharmacists**

This survey, designed by academic professors, aims to examine job satisfaction among community pharmacists and its association with workload, workplace perceptions, and mental health outcomes, including anxiety, depression, and perceived stress.

Your individual privacy will be maintained in all published and written data resulting from the study. Participation is voluntary, and you have the right to withdraw at any time without any consequences.

The survey will take approximately 10 minutes to complete.

Your valuable insights are essential to this study, and we truly appreciate your participation.

Thank you for your time and support.

**I have read instructions and I agree to participate in this study:**

**Yes**

**Sex**

- ☐ Female
- ☐ Male

**Age (years)**

- 22-26
- 27-34
- ≥35

**Years of experience**

- ☐ < 5 years
- ☐ 5–10 years
- ☐ >10 years

**Educational degree**

- ☐ Pharmacy (B Pharm)
- ☐ Doctor of Pharmacy (PharmD)
- ☐ Master’s degree
- ☐ PhD

**Current employment**

- ☐ Full-time
- ☐ Part-time

**Type of pharmacy**

- ☐ Chain
- ☐ Independent

**Work Shifts**

- ☐ Daytime
- ☐ Evening
- ☐ Night
- ☐ Rotating shifts
- ☐ Flexible hours

**Working overtime?**

- ☐ Yes
- ☐ No

**The average number of patients you handled per shift: ___**

**The average number of prescriptions filled per shift: ___**

**The average number of medications you dispensed per shift: ___**

**Are you receiving a rewarding salary?**

- ☐ Yes
- ☐ No

**The job provides sufficient benefits (e.g., medical insurance, retirement plan, etc…)**

- ☐ Yes
- ☐ No

**Feeling the community undervalues the role pharmacists play**

- ☐ Yes
- ☐ No

**Feeling career growth pathways within pharmacy are limited**

- ☐ Yes
- ☐ No

**Believing in the existence of unethical competition from other pharmacists**

- ☐ Yes
- ☐ No

**Perceiving a clear gap between the responsibilities of community pharmacist and the expectations formed in pharmacy school.**

- ☐ Yes
- ☐ No

**Concerned about the increasing number of pharmacy graduates in the country**

- ☐ Yes
- ☐ No

**Are you satisfied with your job as a pharmacist?**

- ☐ Yes
- ☐ No

**Mental Health Assessment**

**Generalized Anxiety Disorder Scale (GAD-7)**

*Over the last 2 weeks, how often have you been bothered by the following problems?*
Response options:
0 = Not at all | 1 = Several days | 2 = More than half the days | 3 = Nearly every day

- Feeling nervous, anxious, or on edge
- Not being able to stop or control worrying
- Worrying too much about different things
- Trouble relaxing
- Being so restless that it is hard to sit still
- Becoming easily annoyed or irritable
- Feeling afraid as if something awful might happen

**Patient Health Questionnaire (PHQ-9)**

*Over the last 2 weeks, how often have you been bothered by the following problems?*
Response options:
0 = Not at all | 1 = Several days | 2 = More than half the days | 3 = Nearly every day

- Little interest or pleasure in doing things
- Feeling down, depressed, or hopeless
- Trouble falling or staying asleep, or sleeping too much
- Feeling tired or having little energy
- Poor appetite or overeating
- Feeling bad about yourself, or that you are a failure or have let yourself or your family down?
- Trouble concentrating on things, such as reading the newspaper or watching television?
- Moving or speaking so slowly that other people could have noticed? Or the opposite, being so fidgety or restless that you have been moving around a lot more than usual?
- Thoughts that you would be better off dead or of hurting yourself in some way?

**Perceived Stress Scale (PSS-10)**

*In the last month, how often have you felt or thought the following?*
Response options:
0 = Never | 1 = Almost never | 2 = Sometimes | 3 = Often | 4 = Very often

- been upset because of something that happened unexpectedly?
- felt that you were unable to control the important things in your life?
- felt nervous and "stressed"?
- felt confident about your ability to handle your personal problems?
- Felt things were going your way
- found that you could not cope with all the things that you had to do?
- been able to control irritations in your life?
- felt that you were on top of things?
- been angered because of things that were outside of your control?
- felt difficulties were piling up so high that you could not overcome them?
- Felt difficulties were piling up too high to overcome
